# Using methylation profiling to diagnose systemic metastases of pleomorphic xanthoastrocytoma

**DOI:** 10.1093/noajnl/vdz057

**Published:** 2019-12-27

**Authors:** Kwok-Ling Kam, Matija Snuderl, Osaama Khan, Jean-Paul Wolinsky, Vinai Gondi, Sean Grimm, Craig Horbinski

**Affiliations:** 1 Department of Pathology, Feinberg School of Medicine, Northwestern University, Chicago, Illinois; 2 Department of Pathology, NYU Langone Health, New York University, New York; 3 Department of Neurosurgery, Feinberg School of Medicine, Northwestern University, Chicago, Illinois; 4 Department of Radiation Oncology, Feinberg School of Medicine, Northwestern University, Chicago, Illinois; 5 Department of Neurology, Feinberg School of Medicine, Northwestern University, Chicago, Illinois

New methods of molecular testing are improving diagnostic accuracy and reliability beyond what the traditional light microscope can provide. In the current case, methylome profiling and next-generation sequencing were used to prove that tumors in the chest wall and spine were metastases from what, by histology, appeared to be a low-grade cerebral glioma. They also identified a targetable BRAF V600E mutation that dictated subsequent therapy, to which the patient responded.

## Case Report

**Fig. 1:**
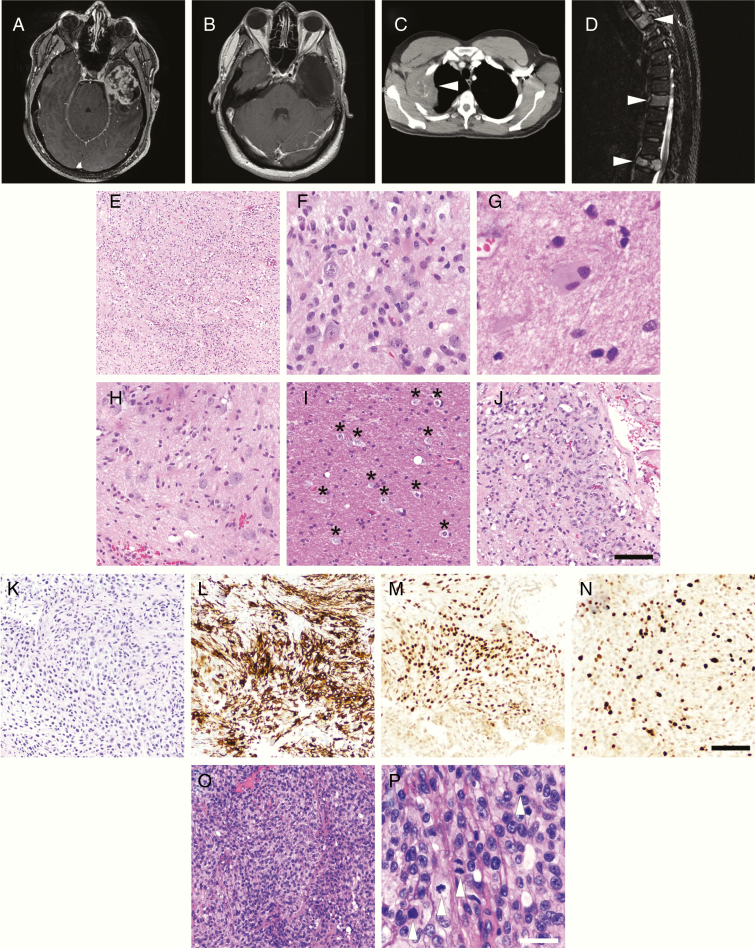
Radiologic and pathologic findings. (A) Post-contrast MRI of the original tumor in the left temporal lobe. (B) Post-contrast MRI of the same site, 2 years later, showing no evidence of local recurrence. (C) MRI of the thorax, showing a mass invading the right chest wall and destroying ribs (arrowhead). (D) MRI inversion recovery sequence of the spine showing vertebral lesions at multiple levels (arrowheads), including a pathologic fracture at T12. The left temporal lobe tumor was of moderate cellularity (E), and had neurons intermixed with bland-appearing spindly-to-round cells (F), as well as rare binucleate neurons suggestive of a ganglioglioma (G). Adjacent cortex had disorganized neurons (H), and there were numerous neurons in the deeper white matter (I, asterisks). No mitoses were present. The herniated area showed microvascular proliferation (J) and necrosis (not shown). Scale bar in J = 250 µm in (E), 50 µm in (F), 25 µm in (G), 100 µm in (H–J). By H&E, the chest wall tumor resembled a sarcoma with a prominent myxoid background (K), yet was immunopositive for the glial markers GFAP (L) and Olig2 (M). A couple of mitoses were seen in the small needle biopsy, and Ki67 was relatively high (N). Scale bar in *N* = 100 µm for (K–N). The T12 vertebral body tumor showed high cellularity (O) and abundant mitoses (P, arrowheads). Scale bar in P = 100 µm in (O), 25 µm in (P).

A 47-year-old man with no prior health issues developed a dull frontal headache that lasted for 1 month. He had no seizures, nausea, vomiting, visual disturbances, or weaknesses. MRI showed a heterogeneous yet fairly well-circumscribed mass in the left temporal lobe that was large enough to produce ipsilateral uncal herniation (Fig. 1A). The tumor underwent gross total resection at an outside hospital. Pathologic evaluation at that outside hospital resulted in an original diagnosis of glioblastoma (GBM). The patient received radiation therapy with concurrent temozolomide (TMZ), but was lost to follow-up for more than 3 months and did not receive post-radiation TMZ. Even after 2 years of follow-up MRIs, there was no evidence of tumor recurrence in the brain (Fig. 1B).

Twenty-seven months after initial presentation, the patient complained of chest and mid-back pain which radiated to the left lumbar area, and difficulty walking. Scans showed a right chest wall mass invading the pleura (Fig. 1C), as well as lesions at multiple levels of the spinal vertebrae, with compressive fracture of T12 causing central canal stenosis (Fig. 1D). The spinal cord and leptomeninges, however, showed no tumors. The patient underwent T1-T4 laminectomy and fusion, T12 vertebrectomy and fusion, and decompression of the spine. A biopsy of the chest wall mass and resection of the T12 mass were performed at the outside hospital and, along with the original left temporal lobe tumor from 2 years prior, were all sent to Northwestern Memorial Hospital for comprehensive review.

## Pathology

**Fig. 2:**
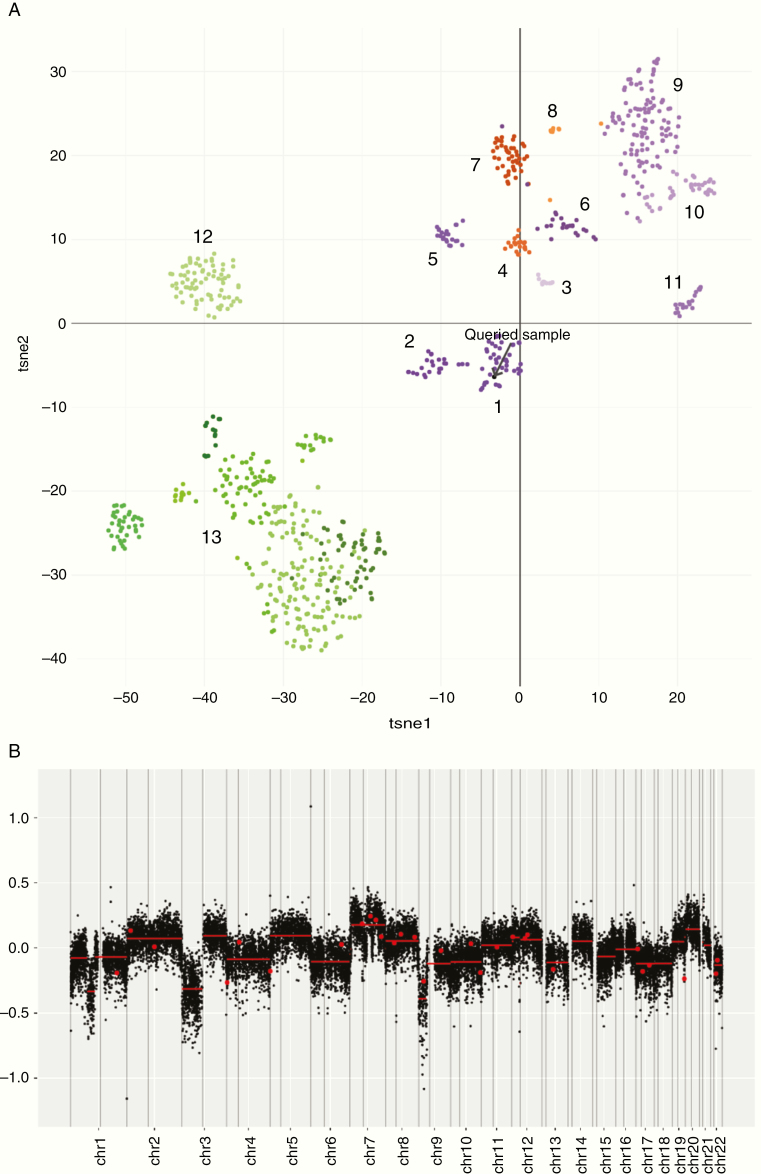
Methylation and copy number profiling of the chest wall mass. (A) The patient’s chest wall mass was plotted on an X–Y coordinate graph (red dot to the lower left of the x–y intersection), where closer proximity to other dots indicates greater similarity of the unknown tumor’s genomic CpG methylation pattern to existing cases in the library. Clusters are as follows: 1 = PXA; 2 = anaplastic PXA; 3 = infantile hemispheric glioma; 4 = ganglioglioma; 5 = *MYB*-driven low-grade glioma; 6 = supratentorial pilocytic astrocytoma; 7 = dysembryoplastic neuroepithelial tumor; 8 = rosette-forming glioneuronal tumor; 9 = posterior fossa pilocytic astrocytoma; 10 = midline pilocytic astrocytoma; 11 = subependymal giant cell astrocytoma; 12 = diffuse midline glioma, K27 mutant; 13 = GBMs (including seven subclusters). Seventy-two tumor clusters, which mapped further away from the queried sample, have been removed for clarity. (B) Data were obtained as part of methylation profiling.

Histologic examination of the temporal lobe resection showed a neoplasm composed of cells with spindly-to-round nuclei (Fig. 1E and 1F). Within the main part of the tumor were neurons and rare binucleate neurons, suggestive of a glioneuronal entity like ganglioglioma (Figs. 1F and 2G). No mitoses were present. Adjacent to the tumor was cortex with dysplastic neurons (Fig. 1H) and abundant neurons in the white matter (Fig. 1I). In the herniated uncinate process, there was necrosis and microvascular proliferation with reactive gliosis and inflammation (Fig. 1J). Neither eosinophilic granular bodies nor Rosenthal fibers were seen.

The T2 chest wall mass showed cells with spindly-to-round nuclei with amphiphilic cytoplasm in an apparently myxoid background (Fig. 1K). Although nuclear atypia was minimal, there were a few mitoses (not shown), the tumor cells were positive for both GFAP (Fig. 1L) and Olig2 (Fig. 1M), and the Ki67 proliferation index was elevated (Fig. 1N). The chest wall mass was immunonegative for CD34, EMA, Ber-EP4, LCA, and Melan-A (not shown).

The T12 tumor showed high cellularity (Fig. 1O), with up to 15 mitoses per 10 high power fields (Fig. 1P) and bone marrow invasion (not shown).

## Molecular Diagnostics

Targeted next generation sequencing (NGS) of the left temporal mass and the chest wall mass showed that both tumors had BRAF V600E mutation, as well as frameshift mutations at L124 in *SETD2* and 124C>T transitions in the *TERT* promoter. The chest wall mass also had an inactivating *TP53* T253A mutation (Table 1).

**Table 1:** NGS comparison of the temporal lobe tumor and the chest wall mass

Temporal lobe tumor	Chest wall mass
BRAF V600E (24.8%)	BRAF V600E (21.5%)
SETD2 L124 (12.0%)	SETD2 L124 (15.0%)
TERT 124 C>T (10.7%)	TERT 124 C>T (12.8%)
	TP53 T253A (16.2%)

Variant allele fraction is shown in parentheses to the right of each mutation.

Methylation profiling of the chest wall mass identified the tumor as a pleomorphic xanthoastrocytoma (PXA, Fig. 2A). The exact score was 0.83 and clustered with PXA reference cases by tSNE analysis. Of note, the random forest classifier and tSNE are independent bioinformatic analyses for evaluation of DNA methylation. The tumor showed widespread copy number alterations, including trisomy 7, but not *CDKN2A* deletion (Fig. 2B). *MGMT* promoter was unmethylated. Considering the overall clinical and radiologic profile, and the histologic similarity to the chest wall tumor, the T12 tumor was interpreted as being part of the same process and was therefore not subjected to further testing. Likewise, the original cerebral tumor was only analyzed by NGS, not methylation profiling, since it contained the exact same mutations as the chest wall mass (aside from the *TP53* mutation).

## Subsequent Clinical Course

After the spinal surgery, the patient developed a pulmonary embolism, prompting placement of an inferior vena cava filter and treatment with apixaban. When the revised molecular-based diagnosis of metastatic PXA with BRAF V600E mutation was rendered, the patient was started on a combination of encorafenib (BRAF inhibitor) and binimetinib (MEK inhibitor). After 3 months of treatment, the main chest wall mass and spinal metastases had shrunk, but additional metastatic disease developed in the lungs and left femoral head. Because of the overall favorable response to BRAF/MEK inhibitors, no radiotherapy to the chest or spine had yet been administered 4 months after chest and spinal surgery.

## Discussion

Advanced molecular tests are not only enhancing our diagnostic capacity, they are also prompting a rethinking of many tumor entities. PXA is an uncommon glial neoplasm; its classic features include location in the superficial temporal lobe, lipid-laden xanthomatous cells, and marked nuclear atypia, including multinucleated cells.^1^ Most PXAs are driven by BRAF V600E mutation,^2^ making it part of the family of brain tumors characterized by MAPK activation. MAPK-driven gliomas are a heterogeneous group of neoplasms histologically and clinically, ranging from dysembryoplastic neuroepithelial tumors to pilocytic astrocytomas (PA) and gangliogliomas (GG), and even a subset of GBMs.^3^ In fact, on consultation, the original cerebral tumor was suspected of being a PA/GG hybrid. As in this case, cortical dysplasia can sometimes be found nearby gangliogliomas and PXAs,^4,5^ further blurring the distinction between developmental malformations and neoplasia.

Because most PXAs are not as infiltrative as diffuse grade II-IV astrocytomas, their prognosis is fairly good when gross total resection is possible. In one retrospective study, 80% of patients with grade II PXAs were still alive after 15 years.^6^ However, grade III PXAs, which are characterized by ≥5 mitoses/high power field, have a median survival of only 5 years.^6^ Grade III PXAs usually also contain other genetic alterations associated with worse behavior, like *TP53* mutations and *TERT* promoter mutations.^3,7,8^ Although the original diagnosis at the outside institution was a GBM, there were no mitoses in the tumor, and the region showing necrosis and microvascular proliferation appeared separate from the main tumor. This was readily attributable to the uncal herniation seen radiologically. Furthermore, unlike a typical GBM, this patient had no recurrences in the brain after 2 years.

Along with BRAF V600E mutation, the original temporal lobe tumor had mutations in the *TERT* promoter and *SETD2;* the latter encodes a histone lysine methyltransferase and has been found most often in high-grade gliomas arising in children and young adults.^9,10^ Even though the original tumor did not appear to be high grade, and the chest wall tumor did not resemble the original tumor, they had the exact same mutations (plus a *TP53* mutation in the chest wall tumor). Thus, NGS definitely showed that the metastatic chest wall tumor came from the original brain tumor, although the pattern of mutations was not specific to a particular type of tumor. GFAP and Olig2 immunopositivity suggested a glioma, but nothing more precise than that.

Methylation profiling by BeadChip array involves obtaining the methylation status of ~450,000 CpG sites (now ~850,000 CpG sites on the most recent EPIC chips from Illumina) and using machine learning to classify new tumors based on overall extent of pattern similarity to existing tumors in the library.^11^ In this case, the original brain tumor showed histologic features more suggestive of PA and/or GG, yet the methylation pattern of its chest wall metastasis unequivocally clustered within the PXA group (Fig. 2A). The distinction between grade II PXA and grade III anaplastic PXA is often difficult histologically, and current methylation classification cannot distinguish between them either. Since the original cerebral tumor showed no histologic evidence of anaplasia, it could be regarded as a grade II PXA, even though its eventual behavior was highly aggressive.

Although rare, extracerebral metastases of PXA have been reported, involving sites as diverse as the scalp, skull, orbit, chest wall, lung, and vertebrae.^12,13^ Vemurafenib, which inhibits BRAF V600E, has shown activity against gliomas with this mutation.^14^ However, since the combination of a MEK inhibitor, binimetinib, with a newer BRAF inhibitor, encorafenib, was shown to be more effective and better tolerated than vemurafenib in BRAF V600E-mutant melanoma,^15^ it was tried in the current case, with good response.

## Funding

This work was supported by National Institutes of Health grant R01NS102669 (C.H.) and by National Cancer Institute P50CA221747.

## Authors’ Contributions

K.K. and C.H. prepared the text and figures. M.S. provided the methylation data. O.K., J-.P.W., V.G., and S.G. edited the clinical portions.

## 

**Conflict of interest statement.** The authors declare that they have no competing interests.
